# Sulfur dioxide alleviates programmed cell death in barley aleurone by acting as an antioxidant

**DOI:** 10.1371/journal.pone.0188289

**Published:** 2017-11-20

**Authors:** Sha-Sha Wang, Ying-Xin Zhang, Feng Yang, Zhong-Qin Huang, Jun Tang, Kang-Di Hu, Hua Zhang

**Affiliations:** 1 School of Food Science and Engineering, Hefei University of Technology, Hefei, China; 2 Xuzhou Institute of Agricultural Sciences of the Xuhuai District of Jiangsu Province, Xuzhou, China; Western Australia Department of Agriculture and Food, AUSTRALIA

## Abstract

Sulfur dioxide (SO_2_), a gaseous signaling molecule in animal cells, has recently been found to play a physiological role in plants. Here we studied the role of SO_2_ in gibberellic acid (GA_3_)-induced programmed cell death (PCD) in barley (*Hordeum vulgare* L.) aleurone layers. The application of the SO_2_ donor (NaHSO_3_/Na_2_SO_3_, 1:3 M/M) effectively alleviated PCD in barley aleurone layers in a dose-dependent manner with an optimal concentration of 50 *μ*M. Further investigations showed that SO_2_ reduced the accumulation of hydrogen peroxide (H_2_O_2_), superoxide anion (⋅O_2_^−^) and malondialdehyde (MDA) in aleurone layers. Moreover, the activities of antioxidant enzymes such as superoxide dismutase (SOD), catalase (CAT), ascorbate peroxidase (APX), glutathione reductase (GR) and guaiacol peroxidase (POD) were enhanced by SO_2_ donor treatment. Meanwhile, lipoxygenase (LOX) activity was attenuated by SO_2_ donor treatment. Furthermore, an induction of endogenous H_2_S and NO were also observed in SO_2_-treated aleurone layers, suggesting interactions of SO_2_ with other well-known signaling molecules. Taken together, we show that SO_2_ negatively regulated PCD by acting as an antioxidant to scavenge excessive reactive oxygen species (ROS) generated during PCD.

## Introduction

Programmed cell death (PCD), a form of cell death initiated and regulated by genes, is a common physiological process during plant development [1]. The cereal aleurone layer is a specialized tissue whose function is to synthesize and secrete hydrolytic enzymes that break down reserves in the starchy endosperm. The process of aleurone cell death is a form of PCD. PCD in cereal aleurone cells occurs after germination, a process that is tightly regulated by gibberellic acid (GA_3_) and abscisic acid (ABA) [2]. Reactive oxygen species (ROS) such as superoxide anion (⋅O_2_^−^), hydrogen peroxide (H_2_O_2_) and hydroxyl radicals are key players in the PCD process in both plant and animal cells [3]. In aleurone cells, ROS, especially hydrogen peroxide, are key players in the hormone-induced PCD [4]. GA_3_ treatment initiates a decrease in the activities of ROS metabolizing enzymes catalase (CAT), ascorbate peroxidase (APX) and superoxide dismutase (SOD) and increases the susceptibility of aleurone cells to oxidative stress [5], suggesting that the reduced ability to scavenge ROS in GA_3_-treated cells may contribute to PCD in aleurone layers.

The gaseous pollutant sulfur dioxide (SO_2_) can be emitted from natural sources, such as microbial and volcanic activities, and by anthropogenic combustion of sulfur-containing fossil fuels. SO_2_ readily hydrates in water to form the sulfite ions, (HSO_3_^1−^ and SO_3_^2−^), strong nucleophiles that can cause damage to a wide variety of cellular components [6]. However, recent findings suggest that endogenous SO_2_ was a novel gasotransmitter in the cardiovascular system and exhibited multiple pathophysiological effects [7]. In plants, exposure to high doses of SO_2_ can cause visible effects including chlorophyll destruction, tissue death and long-term yield reduction [8, 9]. At below toxic levels, plants are able to utilize SO_2_ to satisfy the requirement of sulfur for growth. Indeed, sulfur assimilation and biomass production are correlated with atmospheric SO_2_ which can be reduced to sulfide and further assimilated into cysteine [10, 11]. A recent study found that low concentrations of SO_2_ are able to induce transcriptome reprogramming associated with oxidative signaling and biotic defense responses in plants, suggesting a physiological role for SO_2_ in plants [12]. More evidence indicates that SO_2_/sulfite can induce stomatal closure and promote tolerance to aluminum stress in wheat, and it has been proposed hydrogen sulfide (H_2_S) might mediate the effects of SO_2_ [13, 14].

Like nitric oxide (NO) and H_2_S, SO_2_ can also be produced endogenously from sulfur containing amino acids or sulfate, suggesting that SO_2_ might be a genuine signal in plants [11]. Given the functional similarity between SO_2_, H_2_S and NO in animal cells, we hypothesize that rather than just acting as a harmful gas, SO_2_ might also function as a signaling molecule in delaying GA_3_-induced PCD of barley aleurone layers. Therefore we studied the effect of SO_2_ on antioxidant system to understand the mechanism of the role of SO_2_.

## Materials and methods

### Plant material and treatments

Seeds of barley (*Hordeum vulgare* L.) were kindly supplied by Jiangsu Academy of Agricultural Sciences, Jiangsu Province, China. SO_2_ donor (NaHSO_3_:Na_2_SO_3_, 1:3 M/M) and gibberellic acid (GA_3_) were purchased from Sigma. Seeds were surface-sterilized as described by Chrispeels and Varner [15]. Then the embryo end of seed was removed, and the embryoless half seed imbibed in water at 25°C for 3 days on Petri dishes and culture solutions were renewed every 24 hours. Aleurone layers were gently isolated by scraping away the starchy endosperm with metal spatulas and incubated in a medium containing 10 mM CaCl_2_ and 20 *μ*M GA_3_ with various concentrations of SO_2_ donor (0, 5, 50, 100, 250 or 500 *μ*M) for indicated time. 50 *μ*M SO_2_ donor (12.5 *μ*M NaHSO_3_: 37.5 *μ*M Na_2_SO_3_) was used for parameters determination.

### Cell viability assay in barley aleurone layers

To determine the effect of SO_2_ on cell viability of barley aleurone layers, isolated layers were stained with 0.4% trypan blue [16] for 10 min and photographed with Nikon Eclipse 80i fluorescence microscope (Nikon, Tokyo, Japan). The percentage of dead cells was determined by calculating the percentage of blue or purple cells which indicating dead cells in randomly selected fields from three different aleurone layers per treatment.

### Determination of the contents of superoxide anion, hydrogen peroxide and malondialdehyde

Embryoless half-grains were pretreated with sterile water for 3 d and then the isolated aleurone layers incubated in GA_3_ alone or GA_3_ with 50 *μ*M SO_2_ donor. Contents of ⋅O_2_^–^, H_2_O_2_ and MDA were measured according to the methods in [17]. Three independent experiments with three replicates of 15 half-aleurone layers (0.45 ± 0.001 g) were sampled every 12 h for each treatment.

### Assays of the activities of antioxidant enzymes and lipoxygenase

Activities of superoxide dismutase (SOD, EC 1.15.1.1), catalase (CAT, EC 1.11.1.6), ascorbate peroxidase (APX, EC 1.11.1.11), glutathione reductase (GR, EC 1.6.4.2) and guaiacol peroxidase (POD, EC 1.11.1.7) were determined according to García-Limones et al. [18]. Embryoless half seeds were pretreated with sterile water for 3 days and then incubated in GA_3_ alone or GA_3_ plus 50 *μ*M SO_2_ donor. Frozen aleurone layers (0.45 ± 0.001 g) were homogenized with 1 mL of 200 mM ice-cold phosphate buffer (pH7.8) containing 1.0 mM ethylenediaminetetraacetic acid (EDTA). The homogenate was centrifuged at 12,000 *g* at 4°C for 20 min, and the supernatant was used for antioxidant enzyme activity assay.

Lipoxygenase (LOX, EC 1.13.11.12) activity was determined following the description by Surrey [19]. Samples (0.45 ± 0.001 g) were homogenized with 1 mL of 200 mM phosphate buffer (pH6.0). The homogenate was centrifuged at 15,000 *g* at 4°C for 10 min, and the supernatant was used for activity assay. The assay mixture in a total volume of 3 mL contained 200 mM borate buffer (pH6.0), 0.25% linoleic acid, 0.25% Tween-20, and 50 *μ*L of enzyme extract. The reaction was carried out at 25°C for 5 min, and the activity of LOX was monitored by the changes in absorbance at 234 nm.

### Effect of SO_2_ on the activities of α-/β-amylase

Crude extracts of free and bound β-amylase were prepared according to the method of Guerin et al. [20]. Twenty embryoless half grains (0.7 ± 0.001 g) were homogenized with 10 mL Tris–HCl (50 mM, pH7.5). Then, the homogenate passed through three layers of cheesecloth, and was centrifuged at 10,000 *g* for 30 min. This extraction was repeated three times. The re-suspension of the residue in Tris–HCl buffer was regarded as the bound β-amylase crude enzyme preparation, and the supernatant was collected as free β-amylase crude enzyme. Free form β-amylase was treated with SO_2_ donor at different concentrations (0, 0.01, 0.02, 0.03, 0.04, 0.05, 1.0, 2.0 mM) for 9 h at 4°C. Meanwhile, bound form β-amylase was incubated in 0, 0.1, 0.2, 0.3, 0.4, 0.5, 0.6 or 0.8 mM SO_2_ donor for 9 h at 4°C. To study the effect of SO_2_ to bound β-amylase along with time, 0.8 mM SO_2_ donor was applied to bound form β-amylase for 0, 3, 6, 9 or 12 h at 4°C.

Twenty embryoless half-grains were imbibed in distilled water at 25°C for 3 days on Petri dishes and incubated in Erlenmeyer flasks which contained different concentrations of SO_2_ donor in 20 μM GA_3_ and 10 mM CaCl_2_. Incubation medium was sampled after 24 h and heated at 70°C for 15 min to eliminate β-amylase activity. The activities of β-amylase and α-amylase secreted to the medium were visualized in 10% native PAGE gels by the starch-iodine method according to Collins et al. [21]. To visualize the bands of α-/β-amylase activity, the gel was incubated at 25°C for 30 min in 50 mM PBS (pH7.0) containing 1% boiled soluble starch. After being washed three times with distilled water, the gel was stained with 0.6% I_2_ and 6% KI solution. The experiment was repeated three times and similar results were obtained.

Embryoless half seeds were treated with 20 *μ*M GA_3_ + H_2_O or 20 *μ*M GA_3_ + 1 mM SO_2_ donor and the secreted α-amylase in incubation medium surrounding the half seeds was determined at 0, 12, 24, 36, 48 and 60 h. The DNS method for the determination of secreted α-amylase activity in medium was performed in 0.01 M sodium acetate buffer, pH5.4. The reaction mixture containing 1% soluble starch was incubated at 25°C for 5 min without substrate. Then, the reaction was initiated by adding the substrate and was continued for an additional 10 min at 37°C. The reaction was terminated and hydrolysis was determined with 3,5-dinitrosalicylic acid reagent as modified by Noelting and Bernfeld [22].

### Detection of ROS, H_2_S and NO in aleurone layers by fluorescent probes

Embryoless half seeds were pretreated with sterile water for 3 days. Then aleurone layers were isolated from the embryoless half seeds and were incubated in GA_3_ alone or GA_3_ plus 50 *μ*M SO_2_ donor for 24 and 48 h. Isolated aleurone layers were incubated with the ROS fluorescent probe 2', 7'-dichlorodihydrofluorescein diacetate (DCHF-DA) in 5 *μ*M [23], H_2_S fluorescent probe 3'-methoxy-3-oxo-3H-spiro [isobenzofuran-1, 9'-xanthen]-6'-yl 2-(pyridin-2-yldisulfanyl) benzoate (WSP-1) in 10 *μ*M [24] or NO fluorescent probe 4-amino-5-methylamino-2',7'-difluorofluorescein diacetate (DAF-FMDA) in 10 *μ*M [25] for 20 min at 37°C in the dark according to manufacturer’s instructions. After that, the aleurone layers were washed with distilled water for three times. The fluorescence of DCHF-DA (excitation at 488 nm, emission at 525 nm), WSP-1 (excitation at 465 nm, emission at 515 nm) or DAF-FMDA (excitation at 495 nm, emission at 515 nm) was observed in aleurone layers using a Nikon Eclipse 80i fluorescence microscope (Nikon, Tokyo, Japan). Non-stained aleurone layers were used as negative control. To quantify the intensity of florescence, three different images were analyzed by ImageJ (NIH, Bethesda, Maryland) software, with higher value representing lower intensity of florescence, and vice versa.

### Statistical analysis

Statistical significance was tested by one-way analysis of variance (ANOVA) using IBM SPSS Statistics (SPSS version 20.0; Armonk, NY), and the results were expressed as the means ± SD (standard deviation).

## Results

### SO_2_ donor delays programmed cell death of GA_3_-treated barley aleurone layers

**Fig 1:**
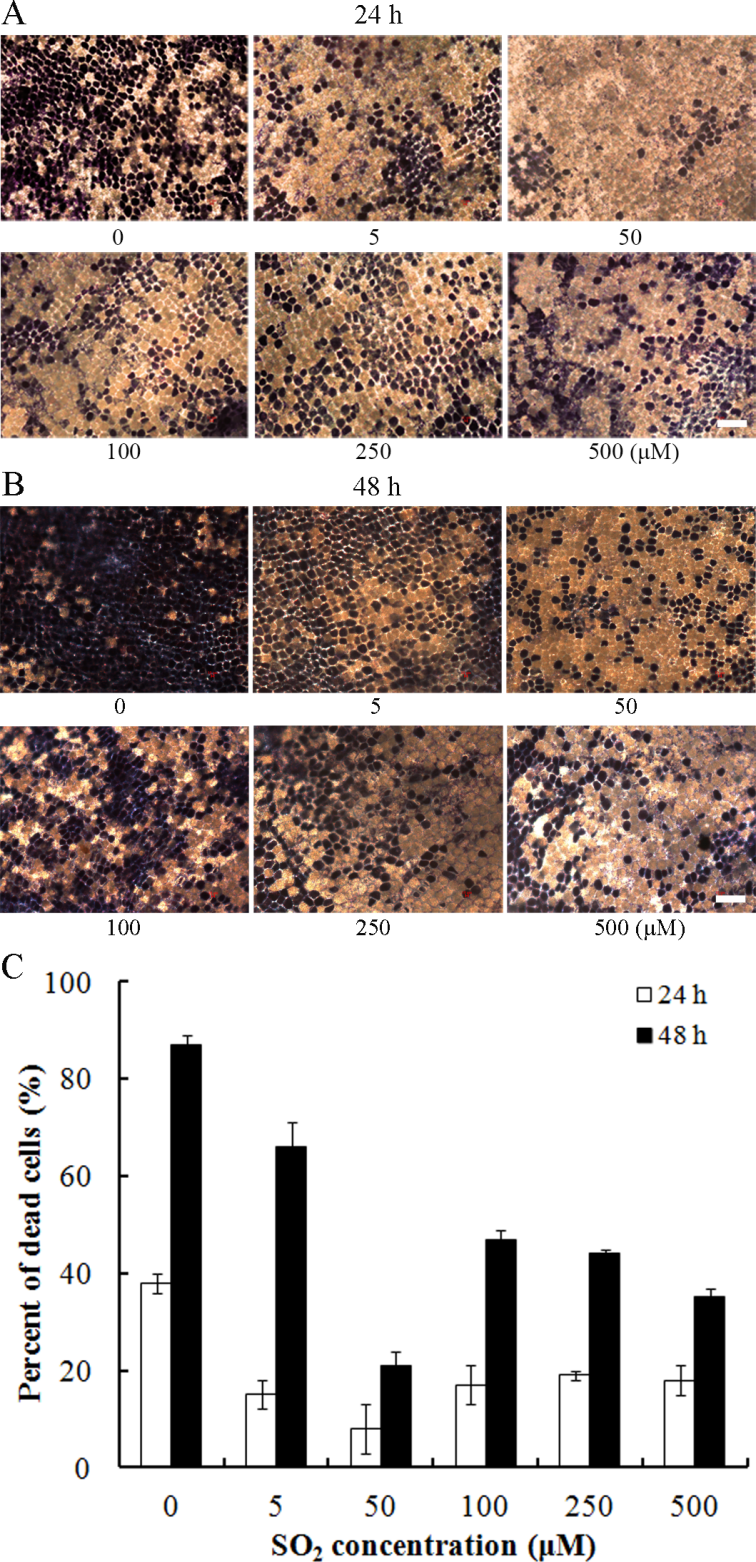
Effect of SO_2_ donor on cell viability in barley aleurone layers. Aleurone layers are incubated in different concentrations of SO_2_ donor (0, 5, 50, 100, 250, and 500 *μ*M) for 24 h (A) and 48 h (B) in presence of 20 μM GA_3_ at 25°C. After staining with trypan blue, barley aleurone layers were viewed by light microscopy. The percentages of dead cells (blue or purple staining) are shown in (C). Bar, 100 μm. Data are expressed as means ± SD of three different aleurone layers per treatment.

Programmed cell death in aleurone layers is stimulated by GA_3_. To study whether SO_2_ is involved in hormonally regulated PCD in aleurone layers, SO_2_ concentrations ranging from 0 to 500 *μ*M was applied to barley aleurone layers subjected to 20 *μ*M GA_3_, and the viability of aleurone cells was monitored at 24 and 48 h (Fig 1). As shown in Fig 1, SO_2_ delayed PCD in GA_3_-treated barley aleurone layers in a dose-dependent manner, with a maximal biological response at 50 *μ*M. After 24 h incubation, 8% of cells were found dead in aleurone layers treated with 50 *μ*M SO_2_ donor, while approximately 38% of cells in control layers underwent PCD (Fig 1A and 1C). After 48 h incubation, the percentage of dead cells increased to 87% in GA_3_-treated aleurone layers in comparison with only a percentage of 21% dead cells in SO_2_ treatment (Fig 1B and 1C).

### Effects of SO_2_ donor on the contents of reactive oxygen species and malondialdehyde in GA_3_-treated barley aleurone layers

**Fig 2:**
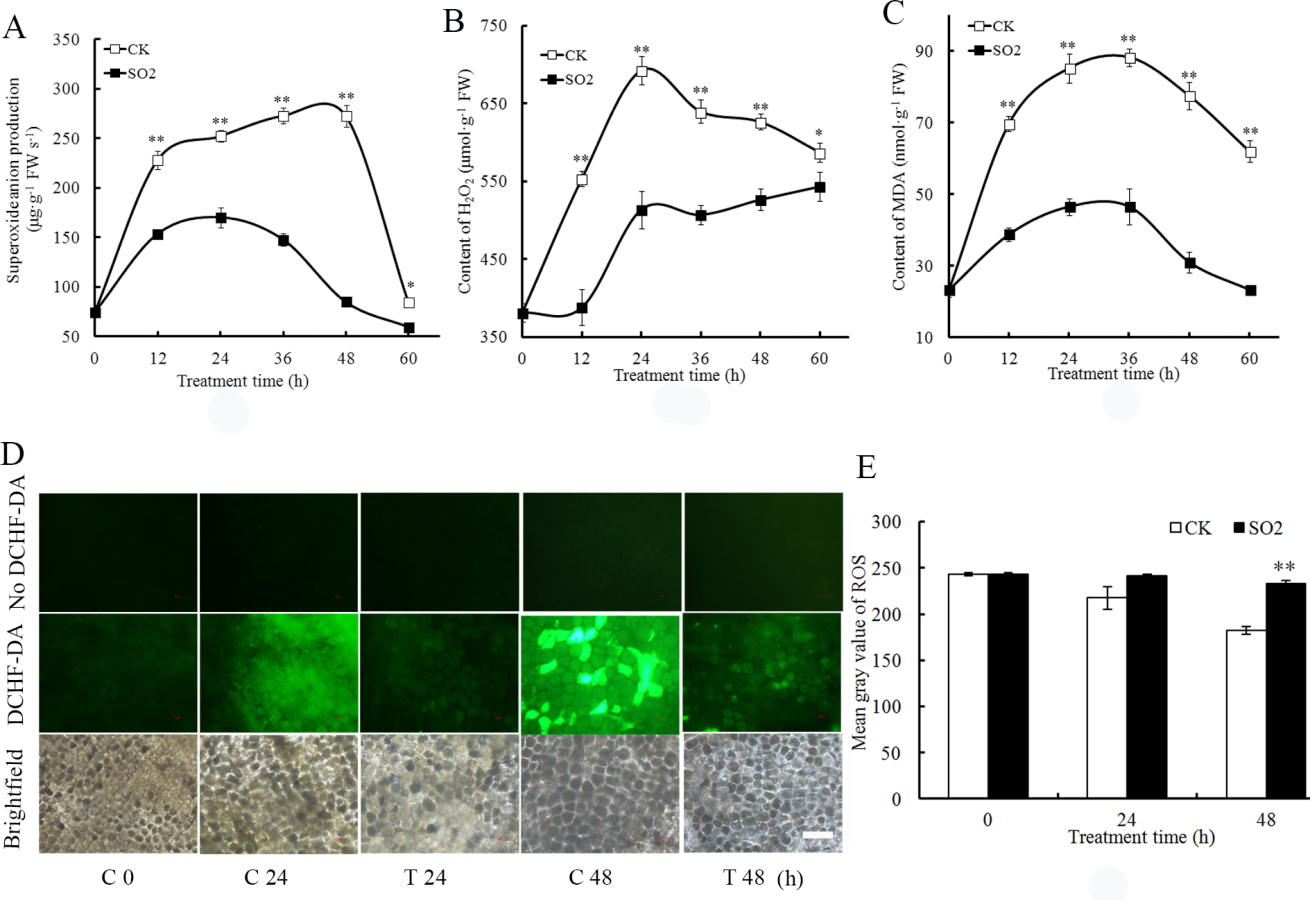
**Effects of SO**_**2**_ **donor on the contents of superoxide anion (⋅O**_**2**_^**–**^**) (A), hydrogen peroxide (H**_**2**_**O**_**2**_**) (B) and malondialdehyde (MDA) (C) in GA**_**3**_**-treated barley aleurone layers.** Aleurone layers are treated with 20 *μ*M GA_3_ + H_2_O (CK) or 20 *μ*M GA_3_ + 50 *μ*M SO_2_ donor (SO_2_) for 60 h. Aleurone layers treated for 24 and 48 h are incubated with DCHF-DA and observed by fluorescence microscopy (D). The relative fluorescence intensity of images analyzed by ImageJ is shown in (E). Data are expressed as means ± SD of three independent experiments with three replicates of 15 aleurone layers per treatment. The symbols * and ** in this figure and following ones stand for significant difference at P < 0.05 and P < 0.01 between the control and SO_2_ treatment, respectively. Bar, 100 *μ*m.

It has been reported that ROS are tightly associated with the promotion of PCD in barley aleurone cells [4]. The alleviating role of SO_2_ in PCD of barley aleurone layers led us to examine whether ROS accumulation was attenuated by SO_2_ treatment. As shown in Fig 2A, the SO_2_ donor significantly alleviated the accumulation of ⋅O_2_^–^ in GA_3_-treated barley aleurone layers. The production of ⋅O_2_^–^ in control aleurone layers increased dramatically during the first 48 h of incubation followed by a decline at 60 h. However the addition of SO_2_ prevented ⋅O_2_^–^ accumulation in aleurone layers. For instance, the production of ⋅O_2_^–^ in SO_2_ treatment at 36 h was about half of that in control layers. Fig 2B illustrates that H_2_O_2_ content in GA_3_-treated layers increased rapidly and peaked at 24 h followed by a gradual decrease. In contrast, the content of H_2_O_2_ in SO_2_-treated layers was maintained at a significantly lower level than that of control as an attenuated accumulation of H_2_O_2_ was observed in SO_2_ treatment (Fig 2B).

MDA was determined as an index of lipid peroxidation. MDA content increased rapidly in control aleurone layers and peaked at 36 h followed by a decrease. In contrast, SO_2_ treatment significantly lowered the level of MDA (Fig 2C).

ROS-sensitive fluorescent probe DCHF-DA was applied to aleurone layers to observe ROS production (Fig 2D and 2E). Fluorescence from layers incubated in SO_2_ plus GA_3_ was much less intense than GA_3_ controls at 24 and 48 h. More weak fluorescence was detected in tissue treated with SO_2_.

### Effects of SO_2_ on the activities of SOD, CAT, APX, GR, POD and LOX in GA_3_-treated barley aleurone layers

**Fig 3:**
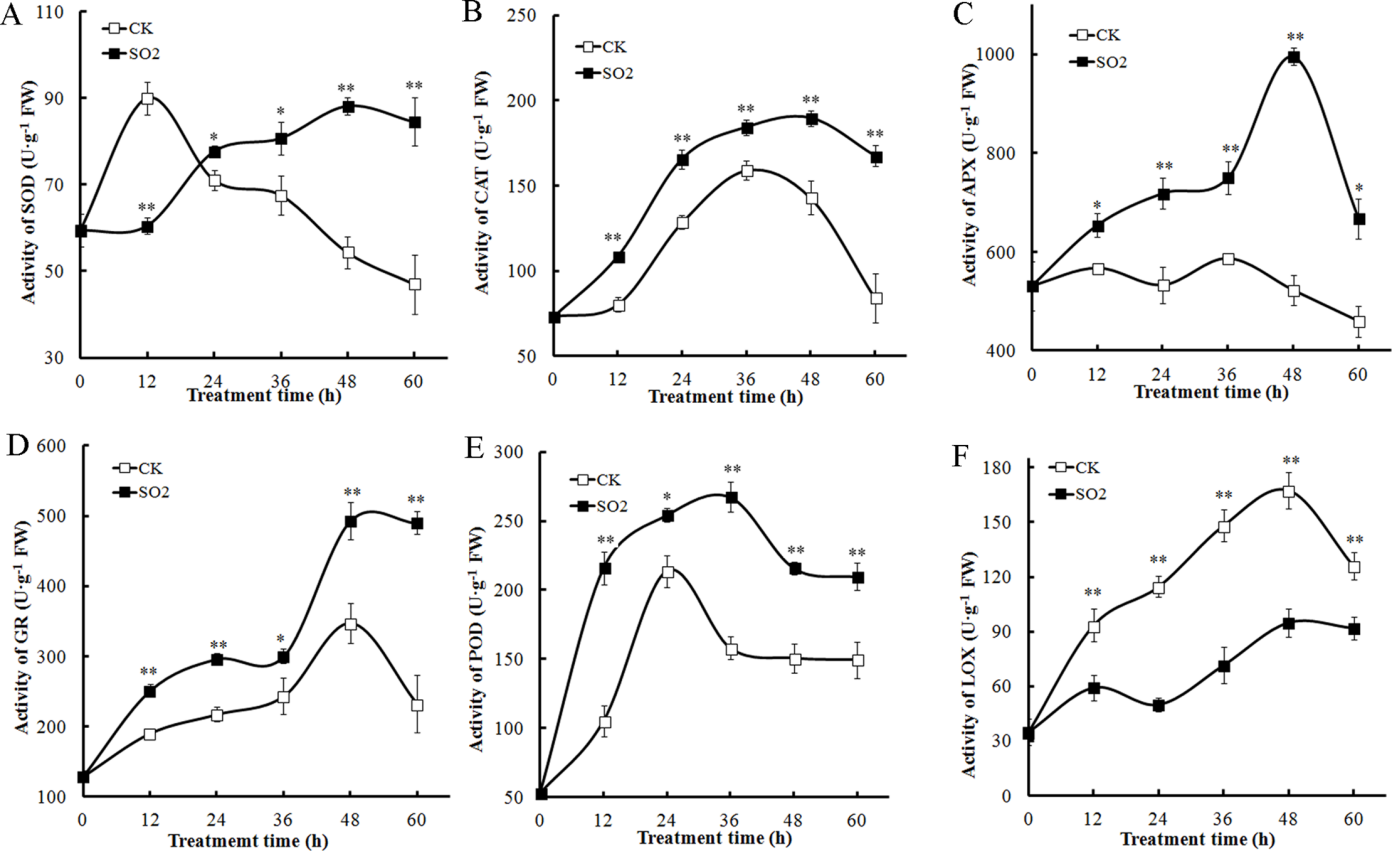
**Effects of SO**_**2**_ **donor on the activities of SOD (A), CAT (B), APX (C), GR (D), POD (E), and LOX (F) in GA**_**3**_**-treated barley aleurone layers.** The aleurone layers were treated with 20 *μ*M GA_3_ + H_2_O (CK) or 20 *μ*M GA_3_ + 50 *μ*M SO_2_ donor (SO_2_) for 60 h. Data are expressed as means ± SD of three independent experiments.

To study the antioxidant role of SO_2_ donor in GA_3_-treated barley aleurone layer, we determined the activities of antioxidant enzymes, such as SOD, CAT, APX, GR and POD, and lipid peroxidation-related enzyme LOX (Fig 3). The activity of SOD increased rapidly in GA_3_-treated aleurone layers in the first 12 h and decreased gradually. However, SOD activity in SO_2_ treatment kept stable during the first 12 h of incubation followed by a steady increase which led to a significantly higher level compared with that of control after 24 h (Fig 3A). As shown in Fig 3B, CAT activity in control increased dramatically till 36 h of incubation followed by a drop. Similar change pattern of CAT activity was observed in SO_2_ treatment, but SO_2_ treatment significantly enhanced CAT activity compared with water control. SO_2_ treatment also significantly improved APX activity in GA_3_-treated aleurone layers during the whole treatment period (Fig 3C). The activity of APX in SO_2_ treatment dramatically increased and peaked at 48 h followed by a sharp decrease. However, APX activity in water control fluctuated and a gradual decrease was observed after 36 h of incubation. GR activity in SO_2_-treated layers increased rapidly during the first 48 h of incubation followed by a plateau, while GR activity in control increased slightly and peaked at 48 h followed by a drop (Fig 3D). During the whole treatment time, SO_2_ significantly enhanced GR activity in barley aleurone layers in comparison to control. Fig 3E shows that the SO_2_ donor maintained a higher level of POD activity in barley aleurone layers compared with control during the entire incubation. POD activity in GA_3_ controls increased and peaked at 24 h followed by a decrease. Compared with GA_3_-treated samples, GA_3_ plus SO_2_ treatment induced a more rapid increase in POD activity until 36 h followed by a decrease (Fig 3E).

LOX activities are responsible for lipid peroxidation. LOX activity increased greatly in GA_3_-treated aleurone layers during the first 48 h followed by a decrease (Fig 3F). By contrast, GA_3_ plus SO_2_ treatment dramatically attenuated the increase in LOX activity though an increasing trend of LOX activity was observed (Fig 3F).

### SO_2_ donor promotes β-amylase activity and α-amylase secretion in GA_3_-treated barley aleurone layers

**Fig 4:**
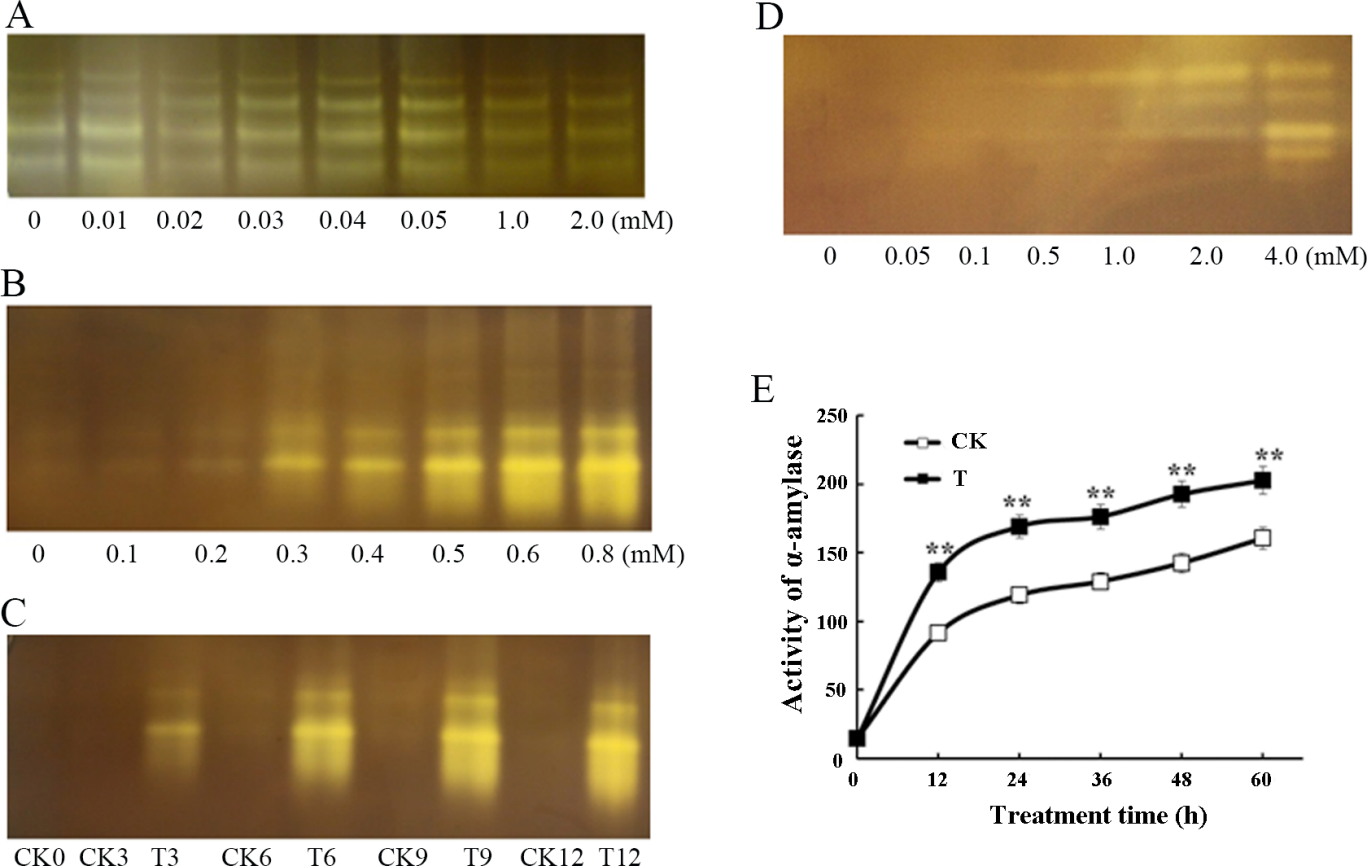
SO_2_ enhances the activities of free and bound forms of β-amylase, and of α-amylase in embryoless barley grains. (A) shows native PAGE analysis of the activity of free form β-amylase in embryoless barley grains treated with different concentrations of SO_2_ donor for 9 h and (B) native PAGE analysis of bound form β-amylase activity. SO_2_ donor at 0.8 mM is applied to bound form β-amylase for 0, 3, 6, 9 and 12 h and the activity is shown on native PAGE (C). Embryoless barley grains are incubated with different concentrations of SO_2_ donor plus 20 *μ*M GA_3_ for 48 h and α-amylase activity in incubation medium surrounding the aleurone layers is visualized by native PAGE (D). (E) shows secreted α-amylase activity in incubation medium surrounding embryoless half seeds treated with 20 *μ*M GA_3_ + H_2_O (CK) or 20 *μ*M GA_3_ + 1 mM SO_2_ donor (T) at different times of incubation. Data in (E) are expressed as means ± SD of three independent experiments with three replicates of 20 embryoless half grains per treatment.

β-Amylase is an exoamylase that hydrolyzes α-1, 4 glycosidic linkages of polyglucan chains at the non-reducing end to produce maltose. Activation of β-amylase by NO was regarded as an early event in seed germination [26]. Thus we examined whether SO_2_ also has a role in β-amylase activation. After treatment of free β-amylase with different concentrations of SO_2_ donor, 0.01 mM SO_2_ donor exhibited a maximal biological response as shown in native PAGE (Fig 4A). Fig 4B shows native PAGE analysis of the activity of bound form β-amylase treated with different concentrations of SO_2_ donor and 0.8 mM SO_2_ donor was found to maximally activate bound β-amylase compared with samples not treated with SO_2_. SO_2_ donor at 0.8 mM was applied to bound form β-amylase for 0, 3, 6, 9 and 12 h to study the time changes of SO_2_ effect (Fig 4C). As shown in native PAGE, treatment of SO_2_ for 9 h effectively activated bound form β-amylase.

In response to GA_3_, α-amylase is secreted from aleurone layers to degrade the starch granules in the non-living endosperm [1]. We therefore tested whether the alleviating effect of SO_2_ on PCD affected the release of α-amylase. As shown in native PAGE of Fig 4D, SO_2_ concentrations above 0.5 mM in the presence of GA_3_ promoted the secretion of α-amylase at 48 h of treatment. Fig 4E shows the time changes in α-amylase secretion from GA_3_-treated aleurone layers and GA_3_ plus 1 mM SO_2_ treated layers. The activity of α-amylase in incubation medium in GA_3_-treated layers increased with time, whereas addition of 1 mM SO_2_ brought about a more rapid increase till 60 h (Fig 4E). The activity of α-amylase released following GA_3_ plus SO_2_ treatment was significantly higher than that of layers incubated only in GA_3_ during the whole treatment time.

### Endogenous H_2_S and NO in SO_2_-treated barley aleurone layers

**Fig 5:**
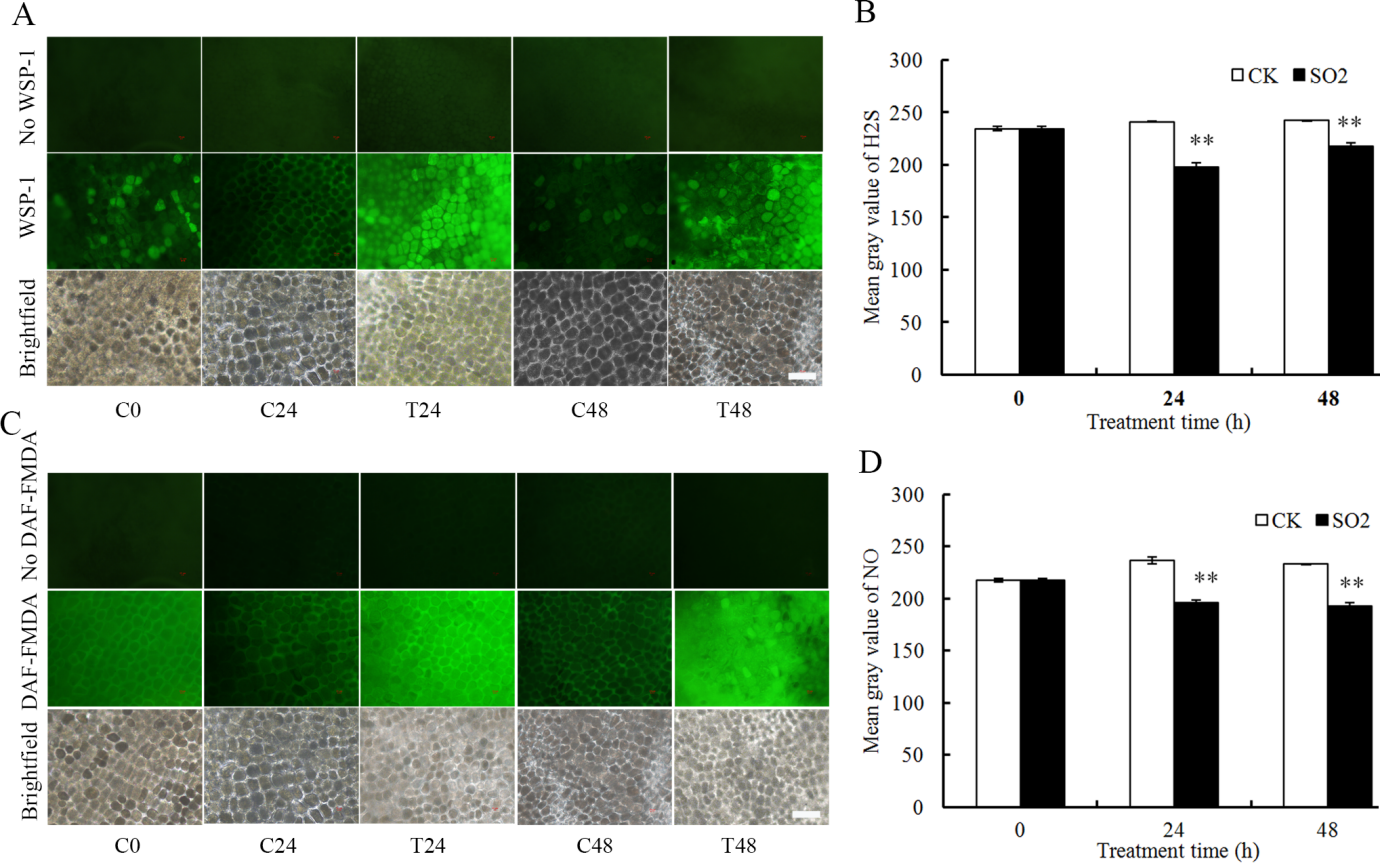
**Effects of SO**_**2**_ **donor on endogenous contents of H**_**2**_**S (A, B) and NO (C, D) in GA**_**3**_**-treated barley aleurone layers.** Microscopy images of barley aleurone layers (A, C) and relative fluorescence intensity of H_2_S (B) and NO (D) analyzed by ImageJ are shown. Barley aleurone layers incubated with the probe WSP-1 (A, B) or DAF-FM DA (C, D) or no probe. CK: 20 *μ*M GA_3_ + H_2_O treatment; T or SO_2_: 20 *μ*M GA_3_ + 50 *μ*M SO_2_ donor treatment. Bar, 100 *μ*m. Data are means ± SD of three independent experiments.

Sulfite can be reduced by sulfite reductase to H_2_S and NO has been found to mediate H_2_S’s function in guard cell movement [11, 27]. To investigate whether exogenous SO_2_ application can induce endogenous H_2_S and NO production, we examined contents of endogenous H_2_S and NO in SO_2_-treated barley aleurone layers. H_2_S in barley aleurone layers was indicated by fluorescent probe WSP-1. As shown in Fig 5A and 5B, SO_2_ treatment significantly enhanced H_2_S fluorescence intensity at 24 and 48 h compared with the weak fluorescence in control. Fig 5C and 5D showed that endogenous NO content in control layers decreased significantly at 24 and 48 h, whereas SO_2_ sustained endogenous NO production.

## Discussion

SO_2_ was regarded as a toxic gas and environmental pollutant. However, SO_2_ can be endogenously generated from metabolism of the sulfur-containing amino acid L-cysteine and from sulfate in plants and its physiological role was recently found in plants [11, 13, 14]. In neural fluid or mammalian plasma, SO_2_ was broken down to its derivatives, bisulfite and sulfite, as the physiological active form of SO_2_ in vivo [28]. Thus NaHSO_3_/Na_2_SO_3_ (1:3 M/M) was chosen as an SO_2_ donor in our study. GA_3_ treatment induced cell death in about 87% aleurone cells at 48 h, while the addition of the SO_2_ donor effectively alleviated PCD process in barley aleurone layers (Fig 1).

ROS, such as ⋅O_2_^−^ and H_2_O_2_, can promote PCD in plant and animal cells [3]. In aleurone cells, GA_3_-stimulated cell death is possibly mediated by ROS while ABA inhibits it [4]. The mechanism of ROS burst is due to GA_3_-caused decrease in the activities of antioxidant enzymes CAT, APX and SOD [1], suggesting that the reduced ability to scavenge ROS in GA_3_-treated cells contribute to PCD. Consistently, the increase of ⋅O_2_^−^ and H_2_O_2_ and the accumulation of MDA were also accompanied by PCD in barley aleurone layers (Fig 2), confirming the vital role ROS played in PCD. However SO_2_ treatment effectively reduced the accumulation of ROS in barley aleurone layers in the presence of GA_3_ (Fig 2), thereby delaying PCD process in these cells. Further investigation indicates that SO_2_ treatment enhanced the activities of ROS-scavenging enzymes SOD, CAT, APX, GR and POD (Fig 3A–3E). The increased activities of ROS-scavenging enzymes in SO_2_+GA_3_ treatment might promote the cell’s ability to scavenge excessive ROS. In addition, LOX activities which are responsible for lipid peroxidation were down-regulated in SO_2_ and GA_3_-treated aleurone layers (Fig 3F).

The role of ROS in GA_3_ and ABA signaling in barley aleurone cells was recently clarified [29]. Furthermore, exogenous H_2_O_2_ could promote the induction of α-amylase by promoting the expression of GAMyb and α-amylase genes, whereas antioxidants suppressed the induction of α-amylase. Unexpectedly, we found that SO_2_ +GA_3_ reduced ROS accumulation and delayed PCD process in barley aleurone layers and meanwhile promoted the secretion of α-amylase (Fig 4D and 4E), suggesting that antioxidants do not always suppress the induction of α-amylase. The effect of SO_2_ on isolated free and bound β-amylase was also researched. SO_2_ treatment significantly enhanced the activity of bound β-amylase and only weakly activated free β-amylase (Fig 4A–4D). The underlying mechanism of SO_2_ in the activation of β-amylase is unknown and still needs further investigation, but the works on NO may shed light on it. As previously reported, NO donor, was able to induce a rapid increase in β-amylase activity by directly releasing β-amylase from its bound form [26].

The sulfite can be reduced by sulfite reductase (SiR; EC 1.8.7.1) by a process that transfers six electrons from ferredoxin to produce the fully reduced sulfide form [11]. H_2_S is already known to function in multiple processes in plants and in some cases, NO mediates the physiological role of H_2_S [27]. Thus the contents of endogenous H_2_S and NO were determined in SO_2_-treated aleurone layers. The increased contents of H_2_S and NO in SO_2_-treated aleurone layers suggest the interplay between sulfite and the two already confirmed signals H_2_S and NO.

## Conclusions

In summary, this data show that the SO_2_ donor alleviates PCD of GA_3_-treated barley aleurone cells by reducing ROS accumulation through enhancing the activities of antioxidant enzymes. Besides, SO_2_+GA_3_ treatment also decreases the activity of LOX, an important indicator of lipid peroxidation. Increased levels of endogenous H_2_S and NO further add more evidence that SO_2_ acts as a novel antioxidant gasotransmitter in PCD of aleurone layers and H_2_S and NO may mediate SO_2_’s role in alleviating PCD.
